# Supplementary material for: A Bayesian method to estimate variant-induced disease penetrance
Source: PLoS Genet. 2020 Jun 22;16(6):e1008862. doi: 10.1371/journal.pgen.1008862 (PMC7347235; doi:10.1371/journal.pgen.1008862)
Supplement: S1 Text — (DOCX) [file pgen.1008862.s001.docx]

# **S1 Text**

We calculated BrS1 “penetrance densities” based on an approach akin to k-nearest neighbors. We average empirical BrS1 mean posterior penetrance ($\frac{BrS1 cases + \alpha_{prior, empirical}}{{{Total carriers + \alpha}_{prior, empirical}+\beta}_{prior,empirical}}$) of variants weighted by the inverse distance in space from the variant of interest. This calculated feature therefore depends on how many variants are near the variant of interest, with regions in three-dimensional space dense with high penetrance variants—“hotspots”—yielding a higher penetrance prediction. Penetrance density is calculated as follows:

$$\rho_{j}=\frac{\sum_{i=0}^{n} {BrS1 Posterior Penetrance}_{i,mutation\left( i\neq j \right)}\cdot\frac{1}{1+e^{(\frac{d_{i,j}}{2})}}}{\sum_{i=0}^{n} \frac{1}{1+e^{(\frac{d_{i,j}}{2})}}}$$

where ρ_j_ is BrS1 penetrance density of the j^th^ variant, *BrS1 Posterior Penetrance_x_*_,i,mutation(i≠j)_ is the empirical BrS1 mean posterior penetrance for the i^th^ variant, and d_i,j_ is the distance between the center of mass of residues i and j. i does include residue j, but only if the identity of the amino-acid mutation is changed, i.e. mutation(i) ≠ mutation(j). For residues in the flexible linkers where structural data are not available, we assumed a flexible amino-acid polymer[1] and calculated the distance between residues as $d_{i,j}=3.8*\sqrt{R_{i,j}}$, where $R_{i,j}$ is the number of residues between residue i and residue j.

1. Smith LJ, Fiebig KM, Schwalbe H, Dobson CM. The concept of a random coil. Residual structure in peptides and denatured proteins. Fold Des. 1996;1(5):R95-106.
