# Supplementary material for: A Bayesian method to estimate variant-induced disease penetrance
Source: PLoS Genet. 2020 Jun 22;16(6):e1008862. doi: 10.1371/journal.pgen.1008862 (PMC7347235; doi:10.1371/journal.pgen.1008862)
Supplement: S2 Text — (DOCX) [file pgen.1008862.s002.docx]

# **S2 Text**

*Determine ‘ν’ from Equation 4.* We scaled the variance from the convergent EM result by a factor of `v`. At each level of `v` and for each variant, we sampled from binomial distribution with n of 100, and probability of $\frac{BrS1 cases+\alpha_{prior, empirical}}{total carriers+{\alpha_{prior, empirical}+\beta}_{prior,empirical}}$. We calculated the resulting 95% posterior credible interval from the Beta distribution with shape parameters 1) ${BrS1 sampled + \alpha}_{prior, EM}$ and 2) ${100-BrS1 sampled+\beta}_{prior, EM}$. We repeated this process 1000 times, and calculated the rate of the posterior credible interval covering the probability $\frac{BrS1 cases+\alpha_{prior, empirical}}{total carriers+{\alpha_{prior, empirical}+\beta}_{prior,empirical}}$ . We selected the best `v` from the coverage plots which balances the tradeoff of over-coverage in variants with medium low BrS1 penetrance and under-coverage of variants with high BrS1 penetrance. From this procedure we found a range of acceptable ‘ν’ values, ‘ν’ = 15-20.
