# Supplementary material for: A Bayesian method to estimate variant-induced disease penetrance
Source: PLoS Genet. 2020 Jun 22;16(6):e1008862. doi: 10.1371/journal.pgen.1008862 (PMC7347235; doi:10.1371/journal.pgen.1008862)
Supplement: S1 Table — (DOCX) [file pgen.1008862.s003.docx]

**S1 Table. *SCN5A* variant-specific features used to predict BrS1 penetrance**

| **Feature** | **Description** |
| --- | --- |
| Peak Current | Maximum sodium conduction, proxy for overall Na_V_1.5 function (protein product of *SCN5A*) normalized to wild type |
| Penetrance Density | Weighted BrS1 penetrance of variants close in space to the variant of interest weighted by the inverse of the distance away.[1] |
| PROVEAN† | Protein Variation Effect Analyzer (PROVEAN) uses pairwise sequence alignment scores to predict functional effects of genetic variants.[2] |
| SIFT† | Sorting Intolerant From Tolerant (SIFT): perturbation predictions based on sequence homology and changes in amino-acid chemistry.[3] |
| PolyPhen-2† | Polymorphism Phenotyping v2 (PolyPhen-2) uses naïve Bayes classification based on a number of predictive features including sequence, phylogenetic and structural information.[4] |
| PAM score† | Point Accepted Mutation (PAM30 matrix) scores amino-acid substitutions based on likelihood of 30 residues per 100 randomly changing over time.[5] |
| Rate of Evolution† | Estimate the rate of evolution per residue using a multiple sequence alignment of homologues and the Rate4site method as previously reported.[6, 7] |
| BLAST-PSSM† | BLAST position specific scoring matrices derived from sequence alignments from the NCBI non-redundant sequence database[8] with PSI-BLAST.[9] |

†Sequence-based feature

1. Kroncke BM, Mendenhall J, Smith DK, Sanders CR, Capra JA, George AL, et al. Protein structure aids predicting functional perturbation of missense variants in SCN5A and KCNQ1. Computational and Structural Biotechnology Journal. 2019;17:206-14.

2. Choi Y, Sims GE, Murphy S, Miller JR, Chan AP. Predicting the functional effect of amino acid substitutions and indels. PLoS One. 2012;7(10):e46688.

3. Kumar P, Henikoff S, Ng PC. Predicting the effects of coding non-synonymous variants on protein function using the SIFT algorithm. Nat Protoc. 2009;4(7):1073-81.

4. Adzhubei IA, Schmidt S, Peshkin L, Ramensky VE, Gerasimova A, Bork P, et al. A method and server for predicting damaging missense mutations. Nat Methods. 2010;7(4):248-9.

5. Schwarz R, Dayhoff M. Matrices for detecting distant relationships. In: Dayhoff M, editor. Atlas of protein sequences: National Biomedical Research Foundation; 1979. p. 353-8.

6. Li B, Mendenhall JL, Kroncke BM, Taylor KC, Huang H, Smith DK, et al. Predicting the Functional Impact of KCNQ1 Variants of Unknown Significance. Circ Cardiovasc Genet. 2017;10(5).

7. Pupko T, Bell RE, Mayrose I, Glaser F, Ben-Tal N. Rate4Site: an algorithmic tool for the identification of functional regions in proteins by surface mapping of evolutionary determinants within their homologues. Bioinformatics. 2002;18 Suppl 1:S71-7.

8. Pruitt KD, Tatusova T, Maglott DR. NCBI reference sequences (RefSeq): a curated non-redundant sequence database of genomes, transcripts and proteins. Nucleic Acids Res. 2007;35(Database issue):D61-5.

9. Altschul SF, Madden TL, Schaffer AA, Zhang J, Zhang Z, Miller W, et al. Gapped BLAST and PSI-BLAST: a new generation of protein database search programs. Nucleic Acids Res. 1997;25(17):3389-402.
