# Supplementary figures and images for: A Bayesian method to estimate variant-induced disease penetrance
Source: PLoS Genet. 2020 Jun 22;16(6):e1008862. doi: 10.1371/journal.pgen.1008862 (PMC7347235; doi:10.1371/journal.pgen.1008862)

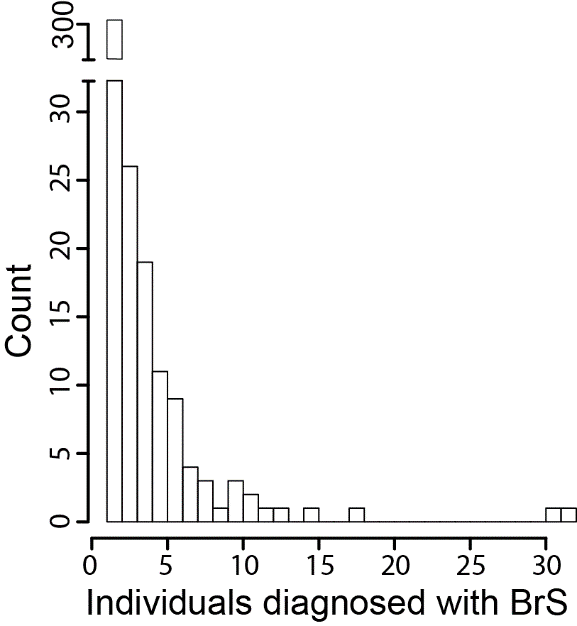

Supplement: S1 Fig — Most variants have only a single heterozygote diagnosed with BrS; however, there are over 10 variants with 10 or more heterozygotes diagnosed with BrS. (PNG) [file pgen.1008862.s005.png]

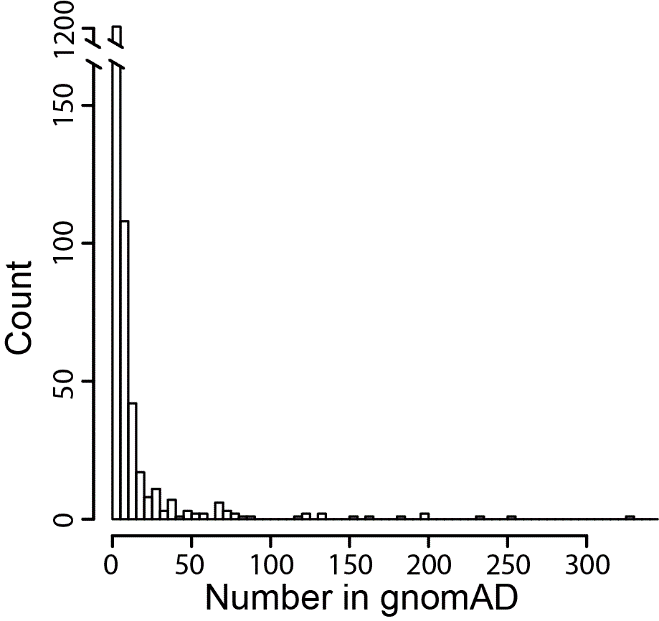

Supplement: S2 Fig — The x-axis is truncated at 350. There are 10 variants with greater than 350 carriers. (PNG) [file pgen.1008862.s006.png]

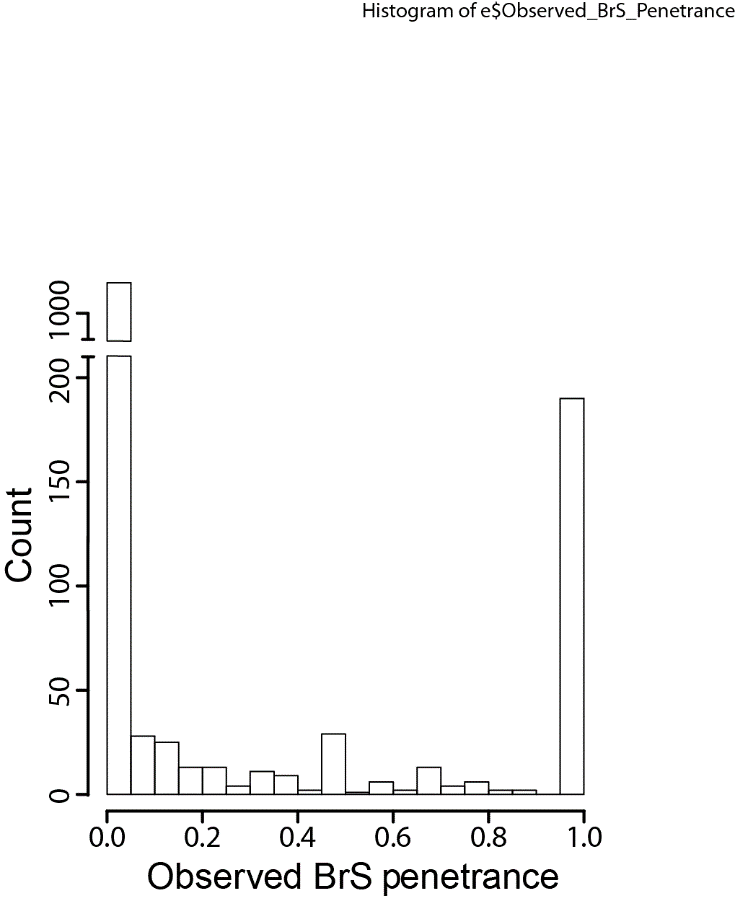

Supplement: S3 Fig — Most variants have either exactly 0 or exactly 1 observed BrS penetrance, at odds with both the known background rate of BrS in the general public (approximately 1 in 10,000–20,000) and with the extreme rarity of any variant having 100% penetrance. (PNG) [file pgen.1008862.s007.png]

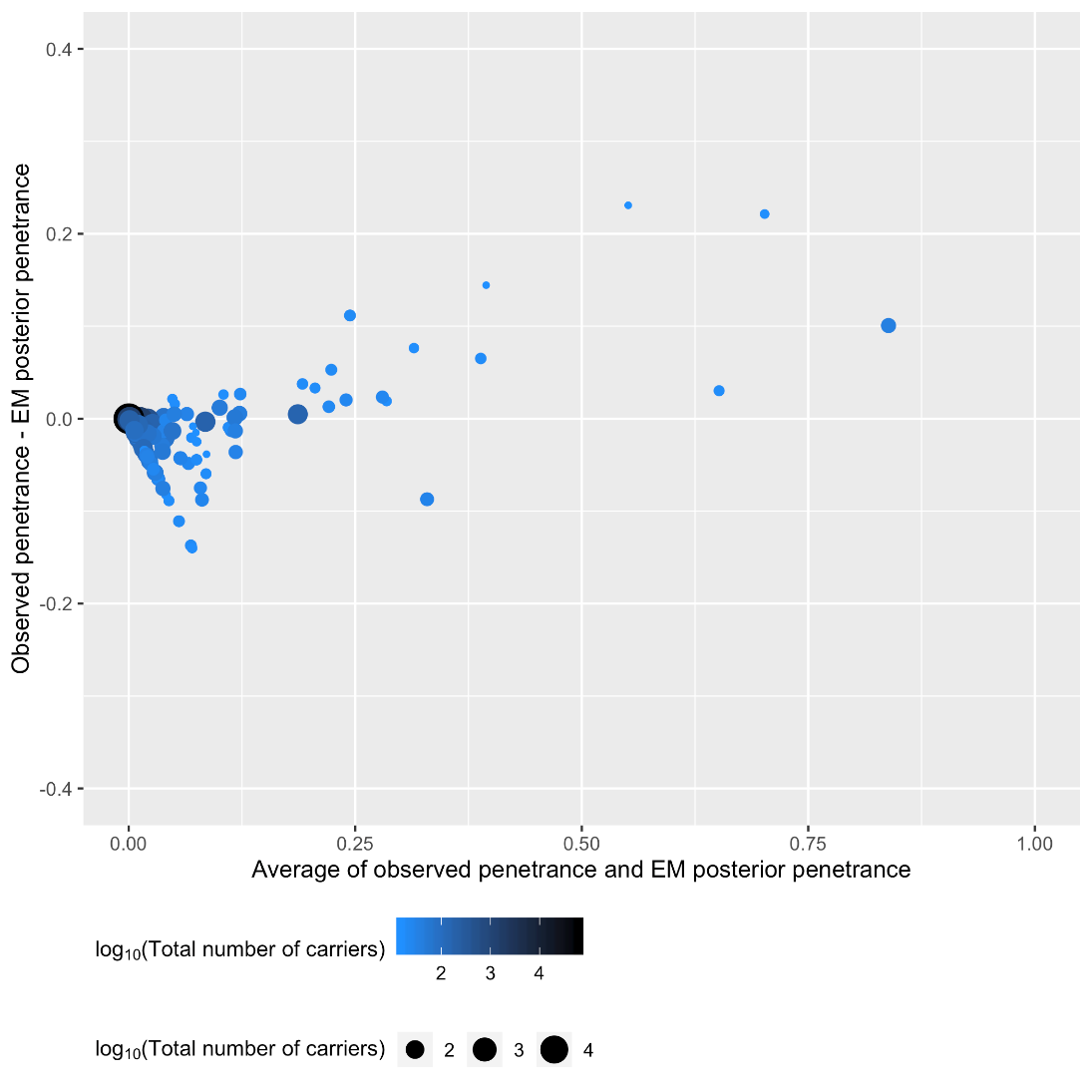

Supplement: S4 Fig — The relatively narrow spread along the y-axis suggests reasonable agreement between the two estimates of BrS penetrance. With the cutoff of at least 15 heterozygotes, there are relatively few variants with an expected penetrance of greater than 10%. (PNG) [file pgen.1008862.s008.png]

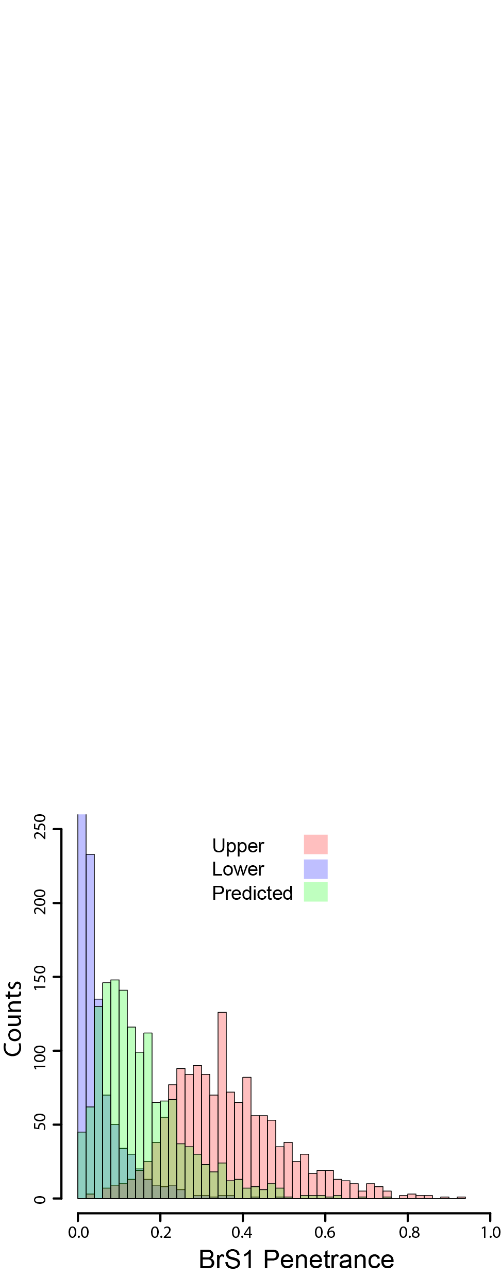

Supplement: S5 Fig — Plotted are BrS1 mean penetrances from imputed EM priors (“Predicted”, green) and upper (red) and lower (blue) bounds to associated 95% credible intervals from those imputed EM priors. (PNG) [file pgen.1008862.s009.png]

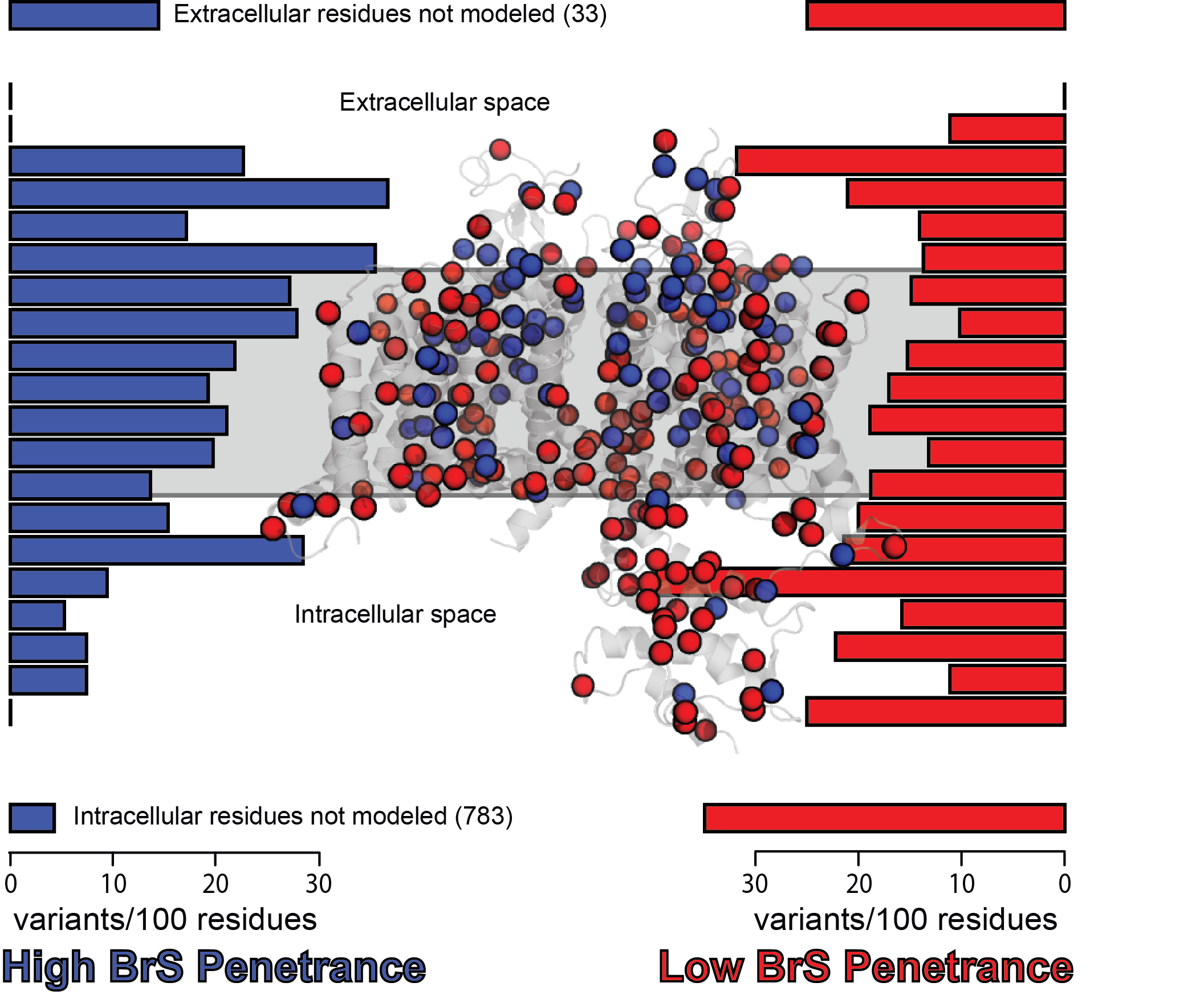

Supplement: S6 Fig — Rate of variants with high BrS1 penetrance (>20%, blue) or low BrS1 penetrance (<10%, red) in a model of the SCN5A protein product. Each bar represents a histogram of variants associated with each disease within a 5Å slice within the membrane (divided by the total number of residues within the slice), boxes at each of the four corners represent residues not modeled (only 33 residues were not modeled in the extracellular loops). There is a relative paucity of low BrS1 penetrance variants within the structured transmembrane region and the relative abundance of high BrS1 penetrance in the same region. The rate of high BrS1 penetrance variants is higher in the extracellular half of the protein molecule likely due to more compacting of residues in the top half of the pore domain as well as proximity to the ion selective element (selectivity filter). Amino acid substitutions in these regions therefore more often have a disruptive influence. (PNG) [file pgen.1008862.s010.png]

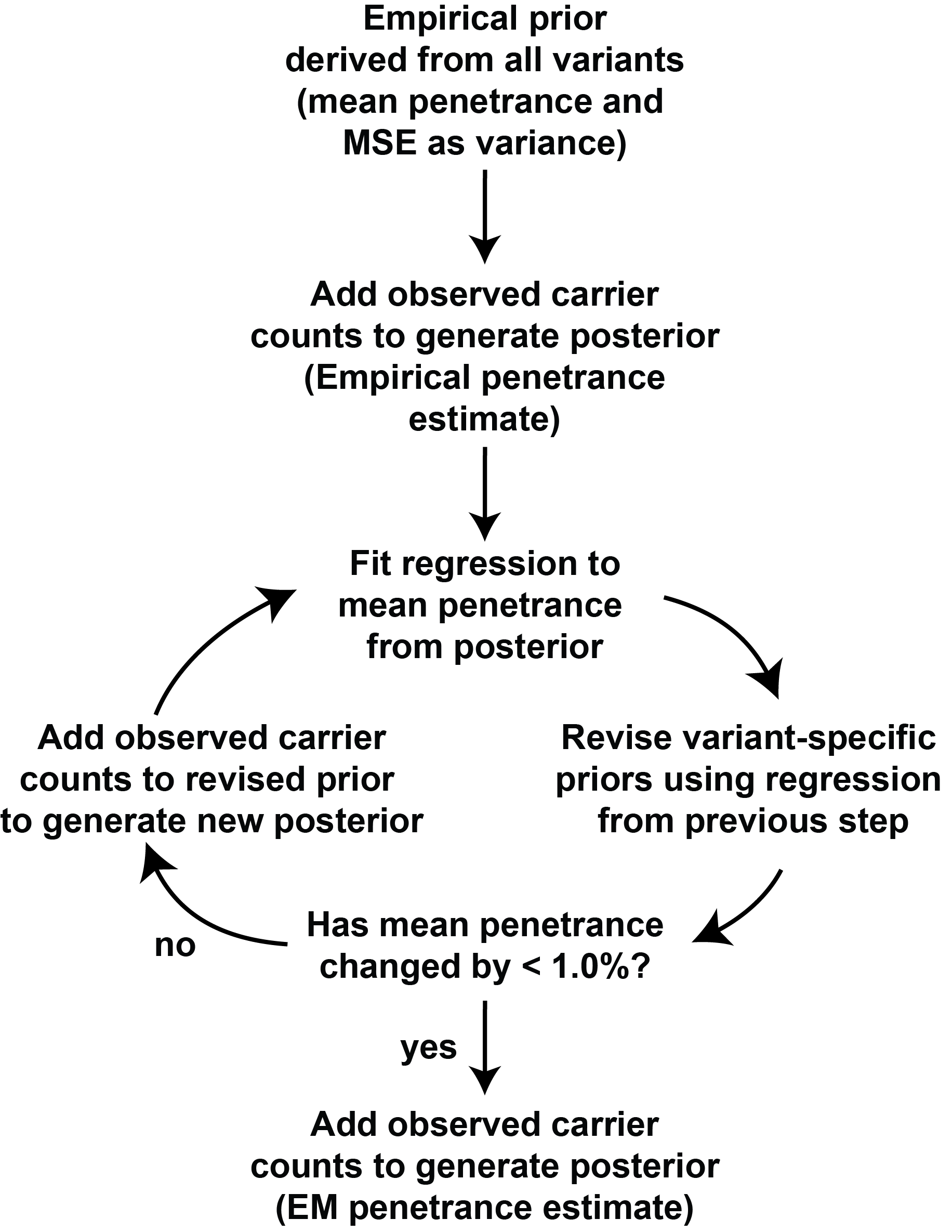

Supplement: S7 Fig — The modified EM algorithm is an iterative technique composed of two steps: 1) calculate the expected penetrance from an empirical Bayes penetrance model and 2) fit regression of our estimated penetrance on variant-specific characteristics by maximum likelihood. The fitted model is then used to generate an updated, imputed prior and subsequent posterior expected penetrance and this process is iterated until it converges to the maximum likelihood solution, when the new mean penetrance changed by less than 1% from the previous iteration. The variance is then estimated according to Eq 4 as explained above. (PNG) [file pgen.1008862.s011.png]

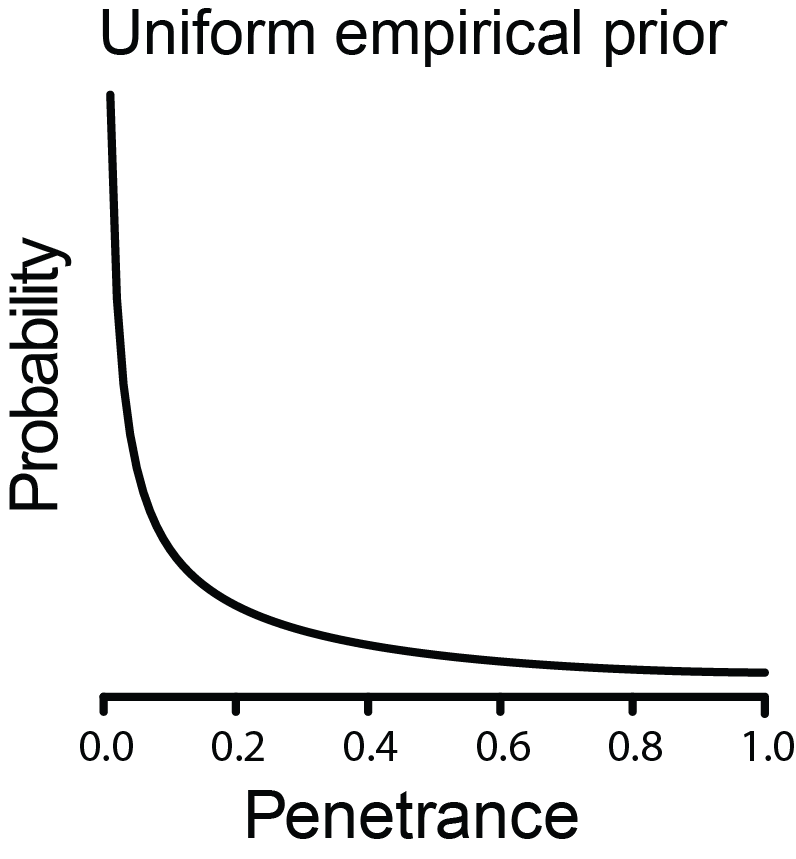

Supplement: S8 Fig — (PNG) [file pgen.1008862.s012.png]

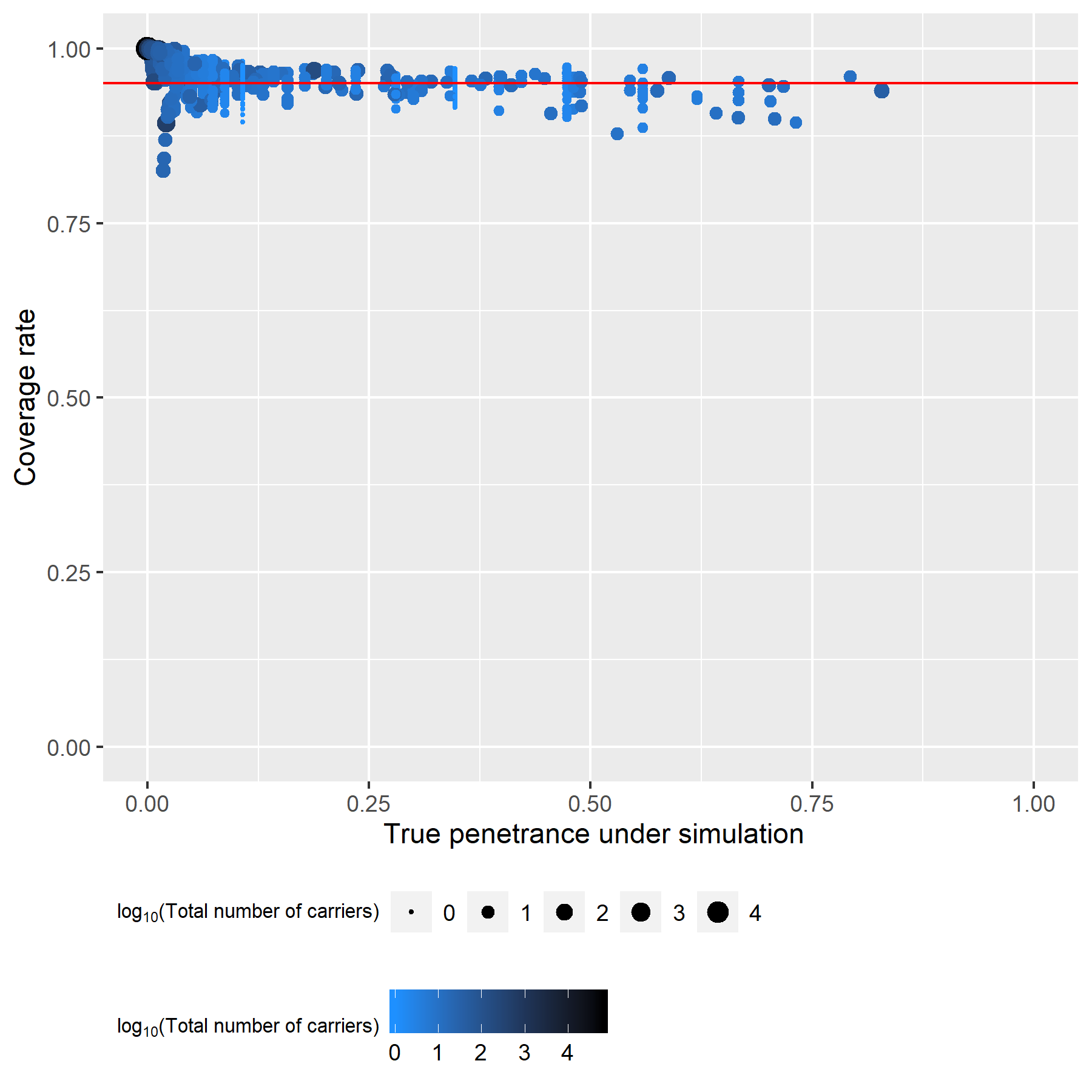

Supplement: S9 Fig — Coverage rate was calculated as defined above. Color and radius indicate the log10 of the total number of heterozygotes present in the dataset. The tuning parameter Eq 4 was set to ν = 7. There is overcoverage (greater than 95%) for variants with high and low BrS1 penetrance indicating an overestimate of the variance. (PNG) [file pgen.1008862.s013.png]

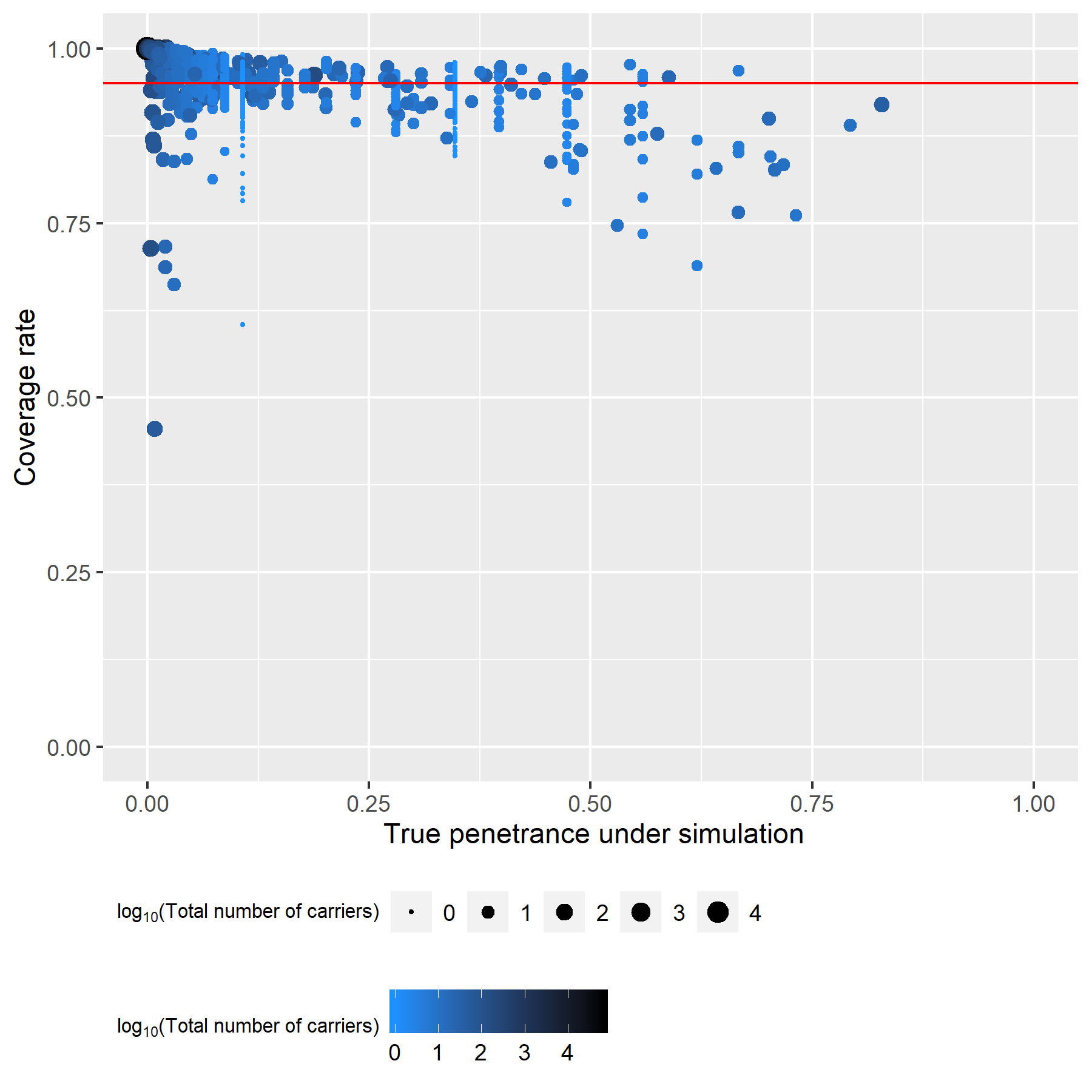

Supplement: S10 Fig — Coverage rate was calculated as defined above. Color and radius indicate the log10 of the total number of heterozygotes present in the dataset. The tuning parameter Eq 4 was set to ν = 14. There is overcoverage for the majority of variants, though some variants are now outside the 95% credible interval. (PNG) [file pgen.1008862.s014.png]

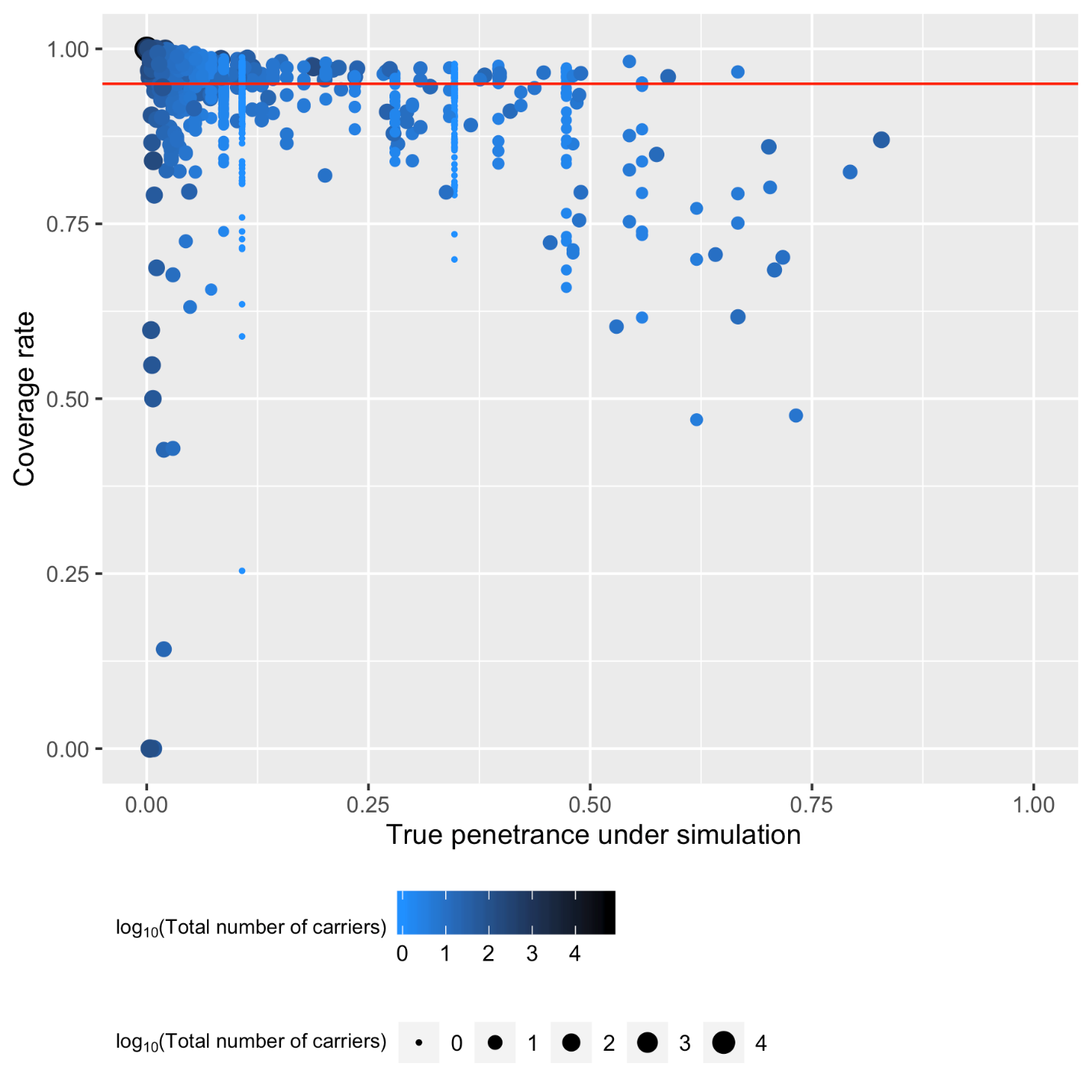

Supplement: S11 Fig — Coverage rate was calculated as defined above. Color and radius indicate the log10 of the total number of heterozygotes present in the dataset. The tuning parameter Eq 4 was set to ν = 19. Overcoverage is reduced especially for residues with very low or very high BrS1 penetrance, indicating an appropriate estimate of variance. (PNG) [file pgen.1008862.s015.png]

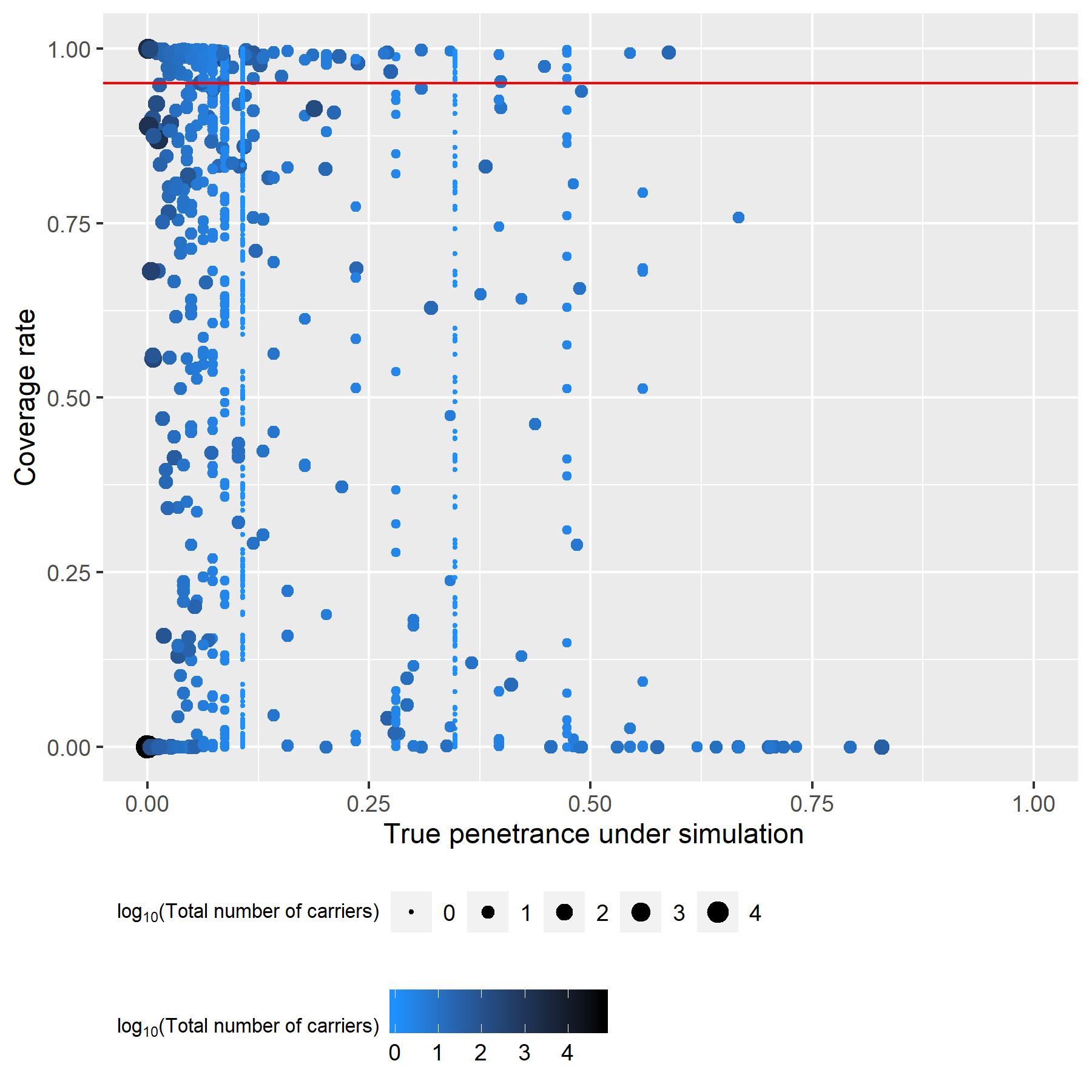

Supplement: S12 Fig — Coverage rate was calculated as defined above. Color and radius indicate the log10 of the total number of heterozygotes present in the dataset. The tuning parameter Eq 4 was set to ν = 99. Variant undercoverage is much more prevalent and distributed evenly across variants with low to high BrS1 penetrance indicating an overestimate of variance. (PNG) [file pgen.1008862.s016.png]
