# Supplementary material for: A Bayesian method to estimate variant-induced disease penetrance
Source: PLoS Genet. 2020 Jun 22;16(6):e1008862. doi: 10.1371/journal.pgen.1008862 (PMC7347235; doi:10.1371/journal.pgen.1008862)
Supplement: Attachment — Submitted filename: Reviews_PLOS_Genetics_v7.docx [file pgen.1008862.s017.docx]

Key:

(Black) Reviewer and Editor comments

(Red) Author responses

(Blue) Specific Revisions to Text

1. You appear to be collapsing the pathogenicity of the variant and the penetrance function of a variant into a single concept. It is not clear to me why this is the correct approach. A VUS can have high (apparent) penetrance and a pathogenic variant could have ~50% penetrance. Your construct would appear to evaluate these the same.

The concepts are closely related, but distinct. The pathogenicity of a variant for a specific individual is binary but unknown; for person X, the variant is either pathogenetic or it is not. The point of this exercise is to reflect the uncertainty about the degree to which this variant will manifest in disease (given the available information at hand). We use the penetrance for this. The penetrance is the probability that the variant will manifest disease for person X; essentially it is a risk score that accounts for all available variant-specific information at the time of assessment.

For example, a VUS might have a 32% penetrance for subjects with a certain predictor profile. We interpret this as meaning that one-third of the patients with this variant will manifest disease. Of course, this assessment is only an estimate, so we spend a considerable amount of effort in the manuscript to provide an uncertainty interval for this estimate: while our best guess for the penetrance might be 32%, the data at hand might also support penetrance in the range of 25% to 40%.

We agree it is important to distinguish between these three concepts: the true but unknown state of nature of the variant for a certain patient (pathogenicity), our best estimate as to whether the variant will result in disease for that patient (penetrance), and the uncertainty in our estimate (the 95% Credible Intervals).

To reflect these changes, we have additionally changed the title to “A Bayesian method to estimate variant-induced disease penetrance”

2. You are inaccurately characterizing the Richards et al and Tavtigian et al heuristics. The former (implicitly) and the latter (explicitly) yield pathogenicity assertions in a nearly continuous probabilistic fashion, but have chosen to use a post-hoc reporting system of five tiers. Anyone using Richards et al can readily see that a variant with one strong and three supporting criteria for pathogenicity is more likely to be pathogenic than would be a variant with one strong and two supporting pathogenic (likely pathogenic criterion iii). You also inaccurately describe this as a three tier system by collapsing P and LP into one category and B and LB into one category and then criticizing it for being too coarse – “…with variants not confidently placed in either of these categories classified as VUS.” Emphasis added. The Tavtigian et al heuristic showed how Richards et al can be transformed into a (nearly) continuous output posterior probability – they are the same. This characterization is also problematic – “Here we advance an approach that quantitates degree of pathogenicity, probabilistically…” but Tavtigian did that as well and you seem to be suggesting that your work in this respect is novel. Your figure 5 is especially problematic in this regard. Even worse that the (incorrect, in my view) collapsing of P & LP, you simply label them both as P.

We have removed figure 5. The intention was to highlight that penetrance is the disease probability conditioned on variant-specific attributes. Our method diverges from Tavtigian because the outcome we are trying to quantitate, penetrance, is probability of disease, not probability of pathogenicity. We have clarified the text to better reflect this distinction. Much of the introduction is rewritten and so we do not reproduce those many changes here.

You should also be aware of a larger issue, which is that there is a strongly held view in medicine that clinicians do not want posterior probabilities at all – they want yes-no answers (you and I don’t accept this view, but I think we are, for the time being, outnumbered). Thus, this is a complicated issue. I think this thrust of your article is not justified and not supported by a review of prior work. Instead, what I think you are doing is describing a novel formulation of a conditional probability and using a different Bayesian formulation than did Tavtigian. That to me seems like a perfectly valid and useful approach and would simplify and clarify your paper.

We agree that a major challenge facing contemporary genomic medicine is the clinical community’s desire (or need) for yes/no answers to the nuanced issue of whether a specific variant will produce a phenotype. That said, some clinical communities do acknowledge probabilities in decision making, e.g. at what probability of predicted coronary artery disease should a statin be started. Additionally, the essential outcome of a diagnostic test is its positive predictive value (PPV) and negative predictive value (NPV), both posterior probabilities, which we have adopted for handling genetic variants. We are proposing a method to estimate a variant-specific PPV (posterior probability of disease, or penetrance) for the diagnostic test of variant discovery. We have removed the quoted passages and adjusted much of the language comparing our penetrance prediction with the ACMG variant annotation guidelines. We rewrote much of the introduction and therefore do not reproduce all changes here.

3. You state that “By quantitatively integrating multiple features, including in vitro functional experiments, information about the three-dimensional protein structure, and previously published variant classifiers, we estimate the BrS1 penetrance attributable to individual SCN5A variants.” This seems to suggest that you are supplanting Richard/Tavtigian PS3 (in vitro functional experiments) PP3 (information about the three-dimensional protein structure) with your measures.

We have modified the language to clarify that we do not suggest these supplant the Richards/Tavtigian classification criteria. We are using these features to estimate BrS penetrance. We include quantitative measures of variant-induced functional perturbation and distances to previously observed residues.

I am troubled by ‘previously published variant classifiers’ – I have no idea what that means.

This is meant to indicate *in silico* predictive methods such as PolyPhen-2 and PROVEAN. We had tried to use the term “*in silico* methods” previously, but we were advised against it since the structural covariates were also produced *in silico*. We have added a hyphen to the term “previously published variant-classifiers” to try and clarify that what we are referencing are variant classification methods that have been previously published. We additionally modified the first mention of previously published variant classifiers:

“previously published variant-classifiers, such as PolyPhen-2 and PROVEAN,”

Later you say this: “Our hypothesis is that features such as variant-induced changes in function, sequence conservation, and location in structure, contain equivalent information to clinically phenotyping carriers and can therefore be used to calculate a penetrance prior. This seems to suggest three criteria, but different than the first three I quote above. The first again seems to be PS3 and are the second and third PP3?

Our point was that information contained in variant-specific features overlaps with information acquired by clinically phenotyping individuals carrying these variants; that penetrance is indicated, to some extent, in variant-specific covariates. We have tried to clarify this language. Given the extensive revision, we do not reproduce all changes here.

4. I am also puzzled as to why this is considered a prior probability. To me, it seems like a conditional probability. You seem to have a very different concept of priors and posteriors than does Tavtigian et al – this needs to be much better explained.

All probabilities are conditional probabilities; what changes are the assumptions and information that they are based on, i.e. the conditioning set changes. Our approach was to use all available information on the penetrance of *SCN5A* variants by incorporating all existing data from past patients and variant attributes into a single probability distribution (our ‘prior’) that reflects our uncertainty about whether this variant is associated with disease or not. This prior is then combined with observed patient data, and results in a posterior probability that the variant will manifest in disease (along with a credible interval). This final posterior probability, which we use as our “risk score”, is essentially a PPV. This is the standard set-up for many diagnostic tests and risk score development. We have updated much of our language in the Introduction and Results/Discussion sections to clarify these points.

5. Your system would not appear to take into account any of the other forms of evidence as does Richards/Tavtigian – why is that? If you do mean to allow those to be taken into account how would that be done? You claim “Our framework captures the same information currently used to adjudicate variants (ACMG guidelines) and reinterprets it quantitatively and probabilistically in terms of risk and uncertainty.” This does not appear to be the case – I do not see in your framework incorporation of the following criteria from Richards et al: PVS1, PS2, PM1, PM2, PM4, PP1, and PP2.

We have tempered much of the comparison of our method, predicting variant-attributable BrS penetrance, and the ACMG guidelines to predict variant pathogenicity. We address each of the listed criteria as follows:

PVS1 states that a mull variant in *SCN5A* is very strong evidence for pathogenicity. We do not treat null variants in our analysis, as they would interfere with the penetrance density calculation. This is clearly an important class of variant, though, for our objectives, they have relatively little variance in the covariates we are trying to leverage to predict penetrance.

PS2 indicates a *de novo* variant found in an affected carrier with no known history of disease is strong evidence for pathogenicity. This evidence assesses the probability a variant is causative for disease in an individual conditioned on the improbability of the coincidence of a spontaneous variant (in a disease-relevant gene) and a spontaneous clinical phenotype (absence of any family history). This evidence may additionally indicate higher marginal penetrance as well and in future work we will investigate how to incorporate this evidence in the estimate of penetrance.

PM4 states that variants which induce protein length changes due to in-frame deletions/insertions in a non-repeat region or stop-loss variants should be considered moderate evidence for pathogenicity. We do not treat in-frame deletions/insertions as a separate category, but include them with missense variants. See more discussion in response to question #2 from reviewer 2.

PP1 indicates cosegregation with disease in multiple affected family members supports classification of pathogenic. This cosegregation is reflected as an enrichment in the disease status of carriers of the variant, which is also reflected in an estimation of penetrance.

PM2 states that the variant is not common enough to preclude a large effect on disease presentation. Our per-variant estimate of disease penetrance includes gnomAD as assumed unaffected carriers such that a variant present in many individuals in gnomAD will have a lower penetrance estimate. Also see response to point #8 below.

PM1 indicates variant location, in relation to other characterized variants, contains information relevant to assigning pathogenicity. Our distance metric, penetrance density, is intended to formalize this intuition by quantitating variant “closeness” to other characterized variants (with estimable BrS penetrance) through three-dimensional space. See also response to point #2 by Reviewer 1.

PP2 indicates missense variants in a gene with a low rate of benign missense variation (and where missense variants are a common mechanism of disease) should be considered supporting evidence for pathogenicity. Our method of estimating penetrance from all variants with known clinical characteristics captures the rate of low and high penetrance variants by leveraging the penetrance data available for many variants, e.g. if all known variants have low or 0 penetrance, the penetrance estimate will also have a low estimate, on average.

We have summarized this as follows:

“*Comparison between penetrance prediction and ACMG variant classification.* We put forward a method to estimate the positive predictive value of discovering an SCN5A variant for BrS1, and uncertainty for that estimate, conditioned on variant attributes. We are not assessing the causality of the variant and its attributes on the manifestation of disease, but rather their association. Hence, our framework diverges from that of the ACMG, quantitated by Tavtigian et al. 2018. For example, in our formulation, a VUS with many affected heterozygotes would have the same probability distribution as a pathogenic variant with many affected heterozygotes (provided the number of observations of cases and controls is the same and the other predictive covariates are the same). If there are comparatively few heterozygotes of the VUS, given the same predictive covariates, the greater uncertainty would be reflected by a wider distribution of penetrance probability (Figure 1). In addition, our calculation is agnostic to origin*, de novo* or inherited, and therefore does not consider this evidence (though this information may additionally inform an estimate of penetrance and therefore warrants further investigation). We also do not treat null variants here. For our purposes of building variant-specific, data-driven penetrance priors, null variants have relatively little variance in the predictive covariates and therefore contribute less to our analysis. In future work we will additionally attempt to include these features.“

6. You state “We suggest the proposed framework is useful in cases both where an individual presents with BrS1 or where a variant is discovered incidentally in an individual not presenting with an arrhythmia.” Are you suggesting that what you describe as ‘penetrance’ – the output of your algorithm is the same in diagnostic or screening contexts? I find that difficult to accept.

The text has been revised. Our approach provides a risk score for disease, in this case BrS1, analogous to a diagnostic test (might patient X develop BrS1 given they have variant Y). If we know patient X already has BrS1, we can use their data to inform other individuals’ risk scores, but we cannot use our approach to absolutely determine the role of variant Y manifesting disease. One application of our approach is that we can examine the ratio P(BrS1|Variant X)/P(BrS1|wild-type) to see if the data better support that variant X is on the causal pathway to disease. But we caution that this approach is imperfect; it does not allow for variants to interact, for example.

“Our approach provides a risk score for disease, in this case BrS1, analogous to a diagnostic test (might patient X develop BrS1 given they have variant Y). If we know patient X already has BrS1, though we can use their data to inform other individuals’ risk scores, we cannot use our approach to absolutely determine the role of variant Y manifesting disease. One application of our approach is that we can examine the ratio P(BrS1|Variant X)/P(BrS1|wild-type) to see if the data better support that variant X is on the causal pathway to disease. But we caution that this approach is imperfect; it does not allow for variants to interact, for example.”

7. What do you define Brugada syndrome to be?

We defined Brugada syndrome by the presence of a spontaneous or drug challenge-induced Brugada syndrome pattern on the ST-segment abnormalities in an ECG. These features are readily identifiable (for the majority of cases) by these electrophysiologic features.^1^ In our literature analysis, we used the assessment of BrS as diagnosed by the clinicians responsible for the report.

We have added the following sentences to clarify this point in the manuscript:

“We define cases as individuals with either a spontaneous or drug-induced ECG BrS1 pattern, ST-segment abnormalities, as reported in each publication.(1)”

We additionally added the following reference:

1.	Mizusawa Y ,Wilde AA. Brugada syndrome. *Circ Arrhythm Electrophysiol*. 2012;5:606-16.

While you roundly criticize Richards et al for being categorical, is it the case that Brugada syndrome is a binary attribute – people either have it or they do not? Given the electrophysiologic nature of this disease, this seems implausible.

BrS has degrees of severity, ranging from intermittent minor ECG abnormalities (so-called type 2 or 3 ECGs) to the more ominous type 1 ECG pattern induced by drug administration or fever to a persistent type 1 pattern to symptoms such as syncope or sudden death. However, we have classified BrS as present/absent because sufficient data are not (yet) reported that would allow us to analyze subtypes.

We clarify this point with the following edits:

“BrS has degrees of severity, ranging from intermittent minor ECG abnormalities (so-called type 2 or 3 ECGs) to the more ominous type 1 ECG pattern induced by drug administration or fever to a persistent type 1 pattern to symptoms such as syncope or sudden death. However, we classified BrS as present/absent due to limited data on subtypes. We envision being able to build a predictive model to predict these degrees of clinical phenotype from variant-specific properties in the future by integrating electronic health records with linked genetic data. At present, these granular electrophysiologic and symptom data are not widely available for a number of unique carriers and unique variants sufficient for statistical analysis.”

But even putting that aside I don’t see how you can define a probability of the penetrance of a disorder if you don’t precisely define what it is. This point seems to be consistent with that of one of the peer reviewers.

We have added greater detail about the diagnosis as used in the manuscript (response to first part of point #7 above). We are modeling the probability of disease (as defined above) given the presence or absence of certain variants and their features.

8. Your assumption that all persons in gnomAD are unaffected is almost certainly wrong. Can you estimate the error introduced by, say 12 people in gnomAD having the disorder? 25 if your estimate of prevalence is off by a factor of 2.0?

To address this concern, we performed a sensitivity analysis. We randomly switched individuals from unaffected (gnomAD) to BrS cases for each variant, and we did this the case of 12, 24, 240, or 2400 misclassified cases. We then re-calculated the variant penetrance of all variants. We repeated this process 1000 times, and checked 1) how often do we see a change in the penetrance, and 2) what is the magnitude of the change (as measured by the mean absolute change).

When the number of misassigned heterozygotes is 12, the median rate of penetrance change is 0.2%, and the expected number of variants with a penetrance change is 4. The average mean absolute difference in penetrance change is 0.01% (first quartile of 1.3e-4, and third quartile of 7e-3).

When the number of misassigned heterozygotes is 24, the median rate of penetrance change is 0.4% and the expected number of variants with a penetrance change is 6. The average mean absolute difference in penetrance change is 0.02% (first quartile of 0.0014% and third quartile of 0.02%).

When the number of misassigned heterozygotes is 240, the median rate of penetrance change is 2%, and the expected number of variants with a penetrance change is 28. The average mean absolute difference in penetrance change is 0.2% (first quartile of 0.1% and third quartile of 0.3%).

When the number of misassigned heterozygotes is 2400, the median rate of penetrance change is 9% and the expected number of variants with a penetrance change is 136. The average mean absolute difference in penetrance change is 2% (first quartile of 2% and third quartile of 2%).

We have also explained these results in the text with the following edits to the methods section:

“Individuals in gnomAD are mostly unaffected, given the rarity of BrS; however, the data available from that resource could be contaminated with individuals presenting with BrS, though likely at or near the rate in the general public. To test the sensitivity of our results to this type of misclassification, we randomly switched individuals from unaffected (gnomAD) to BrS cases for each variant and examined the change in penetrace due to misclassification. We did this with 24 and 240 misclassified cases. With 24 misclassifications, the median rate of penetrance change is 0.4% and the expected number of variants with a penetrance change is 6. The average mean absolute difference in penetrance change is 0.02% (first quartile of 0.0014% and third quartile of 0.02%). With 240 misclassifications, the median rate of penetrance change is 2%, and the expected number of variants with a penetrance change is 28. The average mean absolute difference in penetrance change is 0.2% (first quartile of 0.1% and third quartile of 0.3%). These results suggest minimal influence of small or modest misclassification rates on penetrance estimates.”

9. I do not think that individuals heterozygous for a variant associated with a disorder that is inherited in an autosomal dominant pattern of inheritance should be described as carriers. Better to say ‘heterozygotes’ and reserve ‘carriers’ for disorders inherited in an autosomal recessive pattern.

We have revised the terminology throughout.

10. You should use HGVS nomenclature for all variants. Please specify the cDNA change with the first citation of a variant (subsequent cites of that variant can use protein only, but should use proper protein nomenclature and three letter codes e.g., p.(Arg878Cys).)

We have made the suggested changes

Reviewer's Responses to Questions

**Comments to the Authors:**
Reviewer #1: The authors built a model to predict Brugada Syndrome (BrS1) penetrance of SCN5A variants and assume that the severity of the variant predicts the probability of having disease symptoms (penetrance). The authors have a good idea to move from categorical classification of variants to a more quantitative measure. However, although the gene function and structure are known to predict variant pathogenicity, it is not clear from this manuscript that they predict penetrance as well. Penetrance is much more complicated than just variant pathogenicity for BrS. They have chosen a disease to study that is somewhat complicated.

Major comments:

1. The authors use data from a collection of 756 publications. The studies recruited participants using different criteria. Given the heterogeneity in the clinical characterization of the clinical phenotyping across studies and the difficulty in making the clinical diagnosis of BrS, I am not confident that this is a reasonable data set upon which to build or test a robust model. Furthermore, because the penetrance for BrS is sex and age dependent in addition to modification with fever and medication, the data upon which to base the models are complicated, and the information on the relevant modifiers is rarely published. The details of the dataset should have been summarized in a table describing the clinical cohort derived from the 756 publications along with a figure of the distribution and frequency of the variants studied.

To avoid selection bias, the clinical cohort was drawn from many sources as possible, within reason as per the concerns above. Each individual source was verified to our quality standards, but the reporting of age, sex, ethnicity and other covariates was inconsistent across studies. Of course, there are many genetic modifiers of BrS and an infinite number of environmental factors. Yet there is clearly value in examining the largest data set of BrS cases with variant information that has been published to date. The result of not accounting for additional demographic or environmental factors will no doubt increase the noise in our analysis. To counter this as best as possible, we included the maximum number of carriers for the maximum number of unique variants. The data are now better summarized in the methods section. We additionally include the following figures in the supplement:

“Figure S1: Histogram of the frequency of variants (y-axis) with different number of individuals diagnosed with Brugada syndrome (x-axis). Most variants have only a single heterozygote diagnosed with BrS; however, there are over 10 variants with 10 or more heterozygotes diagnosed with BrS.”

“Figure S2: Frequency of variants (y-axis) with different counts in gnomAD (x-axis). The x-axis is truncated at 350. There are 10 variants with greater than 350 carriers.”

“Figure S3: Frequency of variants (y-axis) with different observed BrS penetrances (x-axis). Most variants have either exactly 0 or exactly 1 observed BrS penetrance, at odds with both the known background rate of BrS in the general public (approximately 1 in 10,000-20,000) and with the extreme rarity of any variant having 100% penetrance.”

2. The model design is not clear. To calculate the prior penetrance, one of the features – penetrance density is based on the observed cases and controls. The posterior penetrance is also calculated based on the observed cases and controls. It is unclear if the convergence criterion is meaningful. What does it optimize?

The EM algorithm is used to provide an estimate of the posterior probability or risk score. We used standard convergence criteria on our objective function (the posterior). To initiate the EM algorithm, we used the data to help specify a reasonable prior, then we derived a posterior from that prior and the data-based likelihood. In practice, that posterior will be used as the prior in another iteration of our algorithm. This is how routine Bayesian updating works. If the concern here is that the data were in some sense used twice to initiate the algorithm, we do not believe that to be a problem because the initial prior is just a starting point. Using a non-informative prior is possible, but it slows down the EM algorithm and is more sensitive to the choice of starting values. We also evaluated two approaches: using a variant-specific (EM) prior or using a common (empirical) prior for all variants. We report on both approaches in the manuscript.

3. How did the authors evaluate the performance of the model? The dataset includes 1000+ variants but only three variants were presented in Figure 1 and 5. The authors used the ClinVar annotation of the three variants to evaluate their estimated penetrance. Pathogenic variants can have penetrance from 0 to 1. The pathogenic variant in the Figure 1 and 5 showed high penetrance, but how do they prove this?

Can the authors compare their posterior penetrance with the observed penetrance when the number of carriers is large enough?

For R878C, shown in Figure 1, we observed 36 individuals who were heterozygote for this variant, 32 of which were diagnosed with BrS1. In Figure 1, we show both the prior (left) and posterior after adding observed data from carriers (right). Additionally, Figure 4 has several variants, 10 of which have 9 or more carriers clinically phenotyped.

We used several metrics to assess the performance of our algorithm at various steps. Overall, a weighted multiple coefficient of determination, R^2^, was computed to summarize the predictive ability of features in each missing data subpattern. Table 1 summaries this for the complete case subpattern. The weighting was done to ensure that variants with greater observations have a greater influence on the final R^2^.

We include Bland-Altman plots showing the difference between the observed BrS penetrance and EM posterior BrS penetrance versus their average for variants with at least 15 heterozygotes. The lack of a systematic pattern is reassuring.

“Figure S#: Bland-Altman plot between EM posterior mean BrS penetrances and observed BrS penetrance for SCN5A variants with at least 15 heterozygotes. The relatively narrow spread along the y-axis suggests reasonable agreement between the two estimates of BrS penetrance. With the cutoff of at least 15 heterozygotes, there are relatively few variants with an expected penetrance of greater than 10%.”

4. From Figure 2, the authors seem to be including common variants in their dataset. These common variants have a BrS penetrance of ~3%, which is too high for common benign variants. This implies the prevalence of BrS is high in their cohort which will inflate the penetrance calculation.

Common variants are included, however their penetrances are near 0 and agreement among estimates is very close for these variants.

5. The prior alpha and beta values depend on the estimated penetrance mean. The authors should not include the benign variants, for which the non-zero penetrance might be due in part to non-genetic reasons or pathogenic variants in other BrS genes.

Benign variants in our data have low to very low penetrance, adjusting the mean lower: not all new variants should be assumed to result in high BrS penetrance because not all known variants result in high BrS penetrance. In fact, rare *SCN5A* variants are approximately 40 times more common than Brugada syndrome itself so we expect many new variants will have little to no influence on BrS penetrance. Additionally, carriers of benign variants were not protected from the disease, so BrS is possible in that genetic context, albeit at a lower penetrance. For these reasons we believe they should be included in the analysis.

Reviewer #2: This paper concerns the widely studied medical genetics problem of classifying variants in disease-causing genes with respect to the potential pathogenicity. The authors develop a Bayesian framework to classify variants on a continuous [0,1] range that represents asymptotic (w.r.t. age) penetrance. They apply this framework to hundreds of variants in the gene SCN5A, which is implicated in Brugada syndrome. The manuscript is well written.

I have only two substantial concerns of the which the first is far more important.

Major concerns.

1. I did not see any pointers to available code or to available posteriors in a form that readers can retrieve. To make the method useful to readers who may be interested in applying the method to
other diseases, the authors need to make their code available. To make the Brugada syndrome analysis useful to medical geneticists who study Brugada syndrome, the results (i.e,, prior and posterior distributions) need to be made available.

All code and datasets are now available on the SCN5A browser website and the Kroncke lab github resources repository.

2. It is unclear how the method handles missing data such as i) variants for which peak current has not been measured or ii) in-frame indels for which several of the sequence analysis tools give no prediction.

To deal with missing data in a prediction model, we followed the approach outlined in Mercaldo and Blume (2018, Biostatistics) with avoids multiple imputation but guarantee maximum predictive accuracy across missing data patterns. In short, for every missing data pattern, we estimate a separate prediction model. To clarify this point, we have added the following text:

”In-frame indels are treated as missense variants. We include these variants as variations at a residue where the indel starts, and only note whether they are an insertion or deletion. Some of these variants have functional data available and their penetrance densities are calculated from the residue starting the indel. These are simplifications to enable an analysis of as many variants and heterozygote individuals as possible.”

”To deal with missing data in a prediction model, we followed the approach outlined in Mercaldo and Blume (42) which avoids multiple imputation but guarantees maximum predictive accuracy across missing data patterns. In short, for every missing data pattern, we estimate a separate prediction model. The models were built with a linear regression pattern-mixture algorithm, updating posterior mean penetrances iteratively until the resulting estimated mean penetrance.”

Minor concerns.

3. The reasoning by which the feature are equivalent to 19 carriers is unclear. It would help to show explicit calculations that explain why 19 is the right number rather than 18 or 20. Moreover, I do not understand why this number does not depend on the number among the 19 phenotyed carriers who turn out to be affected.

This is a tuning parameter. We settled in 19 for these data, and the results were stable after cross-validation. However, this is not a magic number. We examined the uncertainty interval coverage for a given a tuning parameter (ν in equation 4, which is equivalent to the hypothetical number of observations of heterozygotes) over a range (Figures S9-S12). The extremes of ν = 7 and ν = 100 yield a coverage much greater than and much smaller than the expected 95% (since coverage was calculated based on the 95% credible interval of the conditioned prior). ν = 19 seemed to balance both undercoverage at high BrS penetrance and over coverage at low BrS penetrance. Changing this to 18 or 20 would not affect the algorithm much as the results were fairly stable.

In addition, we looked at coverage across variants using forest plots (figure 4) to see if coverage intervals (95% credible intervals) were too wide or too narrow.

Given the comment, we have softened the language in the manuscript to say “around 15-20 carriers” and clarified the text.

4. How are individuals who carry more than one variant treated in the analysis?

We include them as separate records, i.e. two individuals, when these data are available. While we certainly observed this situation, it was very rare. We have indicated this in the manuscript (see response to point #5).

5. How are related individuals carrying the same variant handled in the analysis?

We include both and employ the same diagnostic criteria. Our inclusion criteria are not modified by relatedness.

“For we included compound heterozygotes (individuals with more than one SCN5A variant) as separate records when these data are available though this was very rare. Additionally, our inclusion criteria are not modified by relatedness.”

6. How are unaffected young individuals, who may manifest Brugada syndrome at a later not-yet-reached age, handled in the analysis?

We did not account for the age-dependent penetrance. One could image that all of the controls are actually censored observations, but we felt that this unnecessarily complicates the analysis. We have noted this constraint in the manuscript (See *Limitations*).

7. What is the definition of "match" in the assertion "The penetrance posteriors match classification as presented in ClinVar" in the legend of Figure 1.

We have modified the text to now say “Penetrance priors are low for L1308F (Benign) and higher for R878C (Pathogenic).”

8. alpha and beta have not been defined when they are first used at line 156; they are defined later at line 283

We now define alpha and beta when first introduced.

9. Line 331 refers to "four commonly used servers" but lists only three methods after the colon.

We have adjusted the text to say “three commonly used servers”.

10. The usage of the definition of distance between residues is not clear, especially for in-frame indels and variants in the promoter and splice sites. As an extreme example, it is unclear why the requirement i not equal to j is included in the first big equation in supplemental methods since there could be two distinct mutations affecting the same amino acid, such as p.P1011L and p.P1011S. Adding some examples of the distance calculation involving different
types of variants and unusual situations would help.

We focused our analysis on missense variants as a class, and so compiled predictive covariates (e.g. change in peak current) and clinical phenotypes by variant. We did not consider separating variants based on nucleotide changes that result in the same substitution. This is a good point, however, there are relatively few instances like this. We do not analyze intronic variants here. To clarify this point, we have modified the third paragraph in the materials and methods section.
