# Supplementary material for: A Bayesian method to estimate variant-induced disease penetrance
Source: PLoS Genet. 2020 Jun 22;16(6):e1008862. doi: 10.1371/journal.pgen.1008862 (PMC7347235; doi:10.1371/journal.pgen.1008862)
Supplement: Attachment — Submitted filename: Reviewer critique 2nd-v2.docx [file pgen.1008862.s018.docx]

Reviewer's Questions/Comments

Author Responses

Revisions in Manuscript

This is much improved. One thing I noticed is that the summary of the article needs to be rewritten to shift the narrative to penetrance - it still reads like it is pathogenicity.

We have now changed the summary to “The clinical implications for genetic variants, even definitively pathogenic variants, can vary strikingly across individuals. Lack of evidence to estimate the probability of disease from identified genetic variants, especially rare variants, presents a major barrier to integrating genotype information into clinical care. Here we advance an approach to estimate the penetrance, or positive predictive value of the discovery of a genetic variant, in service of advancing the use of genetic information in personalized medicine.”

Reviewer #1: The authors have dramatically improved the manuscript and addressed most of my previous questions. I still do not agree with including common variants. The common/synonymous variants are very likely to be benign, and it is not useful estimate penetrance for predicted benign variants.

The model we describe translates variant-specific covariates/features into clinically phenotyped individuals heterozygous for those variants. The information they provide is approximately equivalent to the information from 15-20 observations. These variants play a critical role in the calibration of model because we know these variants must have a low penetrance, near or at the general population rate of BrS for genotype-negative individuals. As such, they provide information on predictive value of the covariates/features in our model. Like all regression models, our model ‘borrows the strength’ across penetrance estimates, including common variants, to improve calibration, accuracy, and efficiency. Please also note, when the actual number of observed heterozygotes is much larger than 15-20, our method relies less on this prior information than for when variants where the number of observed heterozygotes is small. Therefore, it is correct that our prior estimates do not modify penetrance estimates of common variants in any substantial way. However, the predictive ability of the covariates/features for these “standard cases” set the baseline for how we interpret rare or yet unobserved variants.

The model can estimate penetrance for a previously variant observed in cases. Penetrance for a new variants (not previously observed) cannot be estimated because you cannot form the likelihood probability. If this is true, this should be stated in the limitations.

We can form the likelihood by borrowing strength in our model. The regression uses the currently fit model to make predictions beyond the observed data, and it assume the basic underlying structure is correct. So, for a variant where any combination of the discussed covariates/features are known (peak current, *in silico* prediction, and structural location), we can estimate BrS penetrance under our model.

Reviewer #2: I mostly limited my assessment of the revision to the question: Did the authors address the 10 comments I made on the initial submission. The authors have not fully addressed my two major comments The authors have fully addressed 6 or 7 of my 8 minor comments. While revising the manuscript the authors introduced some typos and errors in wording.
Major comment 1.
The authors have not addressed my concern about making the code and data available.
I see some files for this project at <https://github.com/kroncke-lab/resources/tree/master/A%20Bayesian%20method%20to%20estimate%20disease%20penetrance%20from%20genetic%20variant%20properties>
However, there is no README file or any other file that I recognize as documentation. The commands git clone <https://github.com/kroncke-lab/resources/tree/master/A%20Bayesian%20method%20to%20estimate%20disease%20penetrance%20from%20genetic%20variant%20properties>
git clone <https://github.com/kroncke-lab/resources/tree/master>
both failed.

We adjusted the GitHub site format to now be more consistent with standard practice. We have updated the README file to better explain the contents of each file.

Major comment 2.
This may be addressed but I do not understand what is a "missing data pattern" and the corresponding new text at lines 405-408. Some examples would help.

We added an example to text to illustrate the pattern mixture method. “For example, His558Arg, where penetrance density, *in silico* predictors, and functional data are all available, the estimate of penetrance is regressed on all other variants where all of these covariates are available (n = 238). For Tyr1449Cys, however, only penetrance density and *in silico* predictors are available, so only those covariates are used in the regression (n = 1,382; much higher since functional data have been collected for relatively few variants).”

Minor comment 3.
I understand the response but did not find where this is explained in the text.

We modified lines 101 and 419 in the current version of the manuscript to remove instances where we mentioned hypothetical carriers of 19 and instead say “interpreted as hypothetical observations of unaffected and affected heterozygotes” and “The resulting priors, imputed from these predictive features, can be readily interpreted as hypothetical observations of unaffected and affected heterozygotes”, at both locations, respectively.

Minor comment 4.
OK
Minor comment 5.
OK, except that the newly added text has a typo.

We have adjusted the text to remove the typo.

Minor comment 6.
The response in place is OK, but I was surprised to see that the authors claimed in response to the Editor that "For person X, the variant is either pathogenic or it is not", which misrepresents the challenge of age-dependent penetrance and this simplistic point of view made it into the revised manuscript at lines 84-85 where the authors wrote "The pathogenicity of a variant for a specific individual is binary, but unknown". The authors cannot have it both ways. Either they acknowledge that age-dependent penetrance is not handled by their method, as suggested by the response to minor comment 6, or they do not.

We do not account for age-dependent penetrance. We acknowledge this point and have modified the referenced line in the manuscript to now say “The pathogenicity of a variant for a specific individual at a given point in time is binary, but unknown. This pathogenicity may have a time dependence such as for diseases which present later in life. Penetrance is one metric that captures the degree to which the pathogenicity will manifest as a human phenotype such as a disease or a trait. We provide posterior probability estimates of the penetrance, asymptotic with respect to age,…”

Minor comment 7.
OK.

Minor comment 8.
OK.

Minor comment 9.
OK.

Minor comment 10.
OK.

Line 373, it is not clear how the indels are scored by the methods such as SIFT; is the score assigned as the worst possible score for any amino acid substitution at the same position?

We do not include *in silico* scores for these variants. We have clarified this with the following:

“For these variants, we did not include *in silico* pathogenicity predictions”

Line 19, "to best assess" is a split infinitive
Lines 105 and 125, change "heterozygotic" to "heterozygous"
Line 109, change "which produce" to "that produce"
Line 172, change "used Bland-Altman plot" to "used a Bland-Altman plot"
Line 272, change "59 genes the ACMG recommends" to "59 genes for which the ACMG recommends"
Line 290, change "The result of not accounting for" to "Not accounting for"
Line 377, change "For we included" to "We included"
Lines 379-380, change "We do not include intronic variant" to "We did not include intronic variants"

We have made the suggested edits.
